# Maternal exposure to metals and persistent pollutants and cord blood immune system biomarkers

**DOI:** 10.1186/s12940-015-0046-3

**Published:** 2015-06-18

**Authors:** Jillian Ashley-Martin, Adrian R. Levy, Tye E. Arbuckle, Robert W Platt, Jean S Marshall, Linda Dodds

**Affiliations:** Interdisciplinary PhD Program, Dalhousie University, IDPhD c/o Faculty of Graduate Studies Room 314 Henry Hicks Building 6299 South St Halifax, Halifax, NS B3H 4H6 Canada; Department of Community Health & Epidemiology, Dalhousie University, Centre for Clinical Research, 5790 University Avenue, Halifax, NS B3H 1 V7 Canada; Population Studies Division, Healthy Environments and Consumer Safety Branch, Health Canada, 50 Colombine Dr., AL 0801A, Ottawa, ON K1A 0 K9 Canada; Department of Epidemiology and Biostatistics, McGill University, Purvis Hall 1020 Pine Ave. West, Montreal, H3A 1A2 QC Canada; Department of Microbiology & Immunology, Dalhousie University Sir Charles Tupper Medical Building, Room 7-C5850 College Street, Halifax, NS Canada; Department of Obstetrics & Gynaecology and Paediatrics, Dalhousie University, Perinatal Epidemiology Research Unit, 7th Floor Women’s Site, IWK Health Centre, 5980 University Ave, PO Box 9700, Halifax, NS B3H 6R8 Canada

**Keywords:** Persistent organic pollutants, Metals, Pesticides, Immunoglobulin E, Thymic stromal lymphopoietin, Interleukin-33, Pregnancy, Cohort

## Abstract

**Background:**

The fetal time period is a critical window of immune system development and resulting heightened susceptibility to the adverse effects of environmental exposures. Epidemiologists and toxicologists have hypothesized that persistent organic pollutants, pesticides and metals have immunotoxic properties. Immunotoxic effects may manifest as an altered immune system profile at birth. Immunoglobulin E, thymic stromal lymphopoietin (TSLP), and interleukin-33 (IL-33) may be implicated in the etiology of childhood allergy and are detectable at birth. The objective of this study was to examine the potential relationship between maternal concentrations of metals, persistent organic pollutants, and pesticides and elevated umbilical cord blood concentrations of IgE, TSLP, and IL-33 in a Canadian birth cohort.

**Methods:**

This study utilized data collected in the Maternal-Infant Research on Environmental Chemicals (MIREC) Study, a trans-Canada cohort study of 2,001 pregnant women. Of these women, 1258 had a singleton, term birth and cord blood sample. Logistic regression was used to determine associations between log-transformed continuous variables and immune system biomarkers.

**Results:**

Inverse relationships were observed between lead, DDE, PCB-118, and a summary index of organophosphorous metabolites and jointly elevated concentrations of IL-33 and TSLP. None of the environmental contaminants were associated with increased odds of a high cord blood immune system biomarker concentration.

**Conclusions:**

In this primarily urban Canadian population of pregnant women and their newborns, maternal blood or urine concentrations of persistent organic pollutants, pesticides, and metals were not associated with immunotoxic effects that manifest as increased odds of elevated concentrations of IgE, TSLP or IL-33.

## Background

In utero exposures to certain environmental contaminants can trigger permanent, irreversible changes to the developing immune system. These changes may promote the persistence of skewed immune system responses and subsequent increased risk of an allergic phenotype [1–3].

Toxicologists and epidemiologists have hypothesized that metals [1, 4, 3], pesticides [5, 6], and polychlorinated biphenyls (PCBs) [7, 8] have immunotoxic properties. The majority of North Americans have detectable blood concentrations of metals [9, 10]. Organophosphate pesticide exposure is common, even in urban populations, due to ingestion of pesticide residues on treated food [11] and presence in household dust [12]. Many adults have detectable concentrations of persistent organic pollutants (POPs), including polychlorinated biphenyls (PCBs) [13] and certain organochlorine pesticides [14] due to their lipophilic and persistent nature [10].

In utero environmental contaminant exposure may be associated with altered levels of immune system biomarkers at birth and possible subsequent risk of childhood allergy [15, 16]. The immune system biomarkers, immunoglobulin E (IgE), interleukin-33 (IL-33) and thymic stromal lymphopoietin (TSLP), have been detected at birth [17] and are integral in the mechanisms underlying allergic responses [18]. Cord blood IgE concentrations have been previously used as a biomarker of the immunotoxic effects of in utero environmental contaminant exposure [19–22]. TSLP and IL-33 have recently been recognized for their etiologic role in atopic dermatitis, the earliest manifestation of childhood allergy [23]. TSLP has also been recently recognized for its role in childhood allergy [24]. Unlike lymphopoietic cell- derived Th2 cytokines, TSLP and IL-33 are produced by epithelial cells and, therefore, not dependent on the presence of a mature immune system [25]. Moreover, epithelial cells have recently been recognized for their role in the regulation of innate and adaptive immune system responses [26, 27]. By lining the digestive, respiratory tract, and skin, epithelial cells may represent the point of entry for environmental contaminant exposure [28].

The objective of the present study was to determine the association between maternal concentrations of pesticides, PCBs, and metals and elevated cord blood concentrations of IgE, TSLP, and IL-33 using data from a Canadian birth cohort. A secondary objective was to assess whether these associations differed by infant sex [29].

## Methods

### Study population

Data and biospecimens were obtained from the Maternal-Infant Research on Environmental Chemicals (MIREC) Study, a trans-Canada cohort study of 2,001 pregnant women from ten Canadian cities recruited between 2008 and 2011 [30]. The target population and exclusion criteria for the MIREC study and present analysis have been described previously [17, 30]. Cord blood samples that were determined to be contaminated with maternal blood based on an elevated immunoglobulin A (IgA) concentration (≥10 μg/mL) were excluded from the analysis [31]. As pre-term and multiple birth infants had lower cord blood samples of the immune system biomarkers and may have a differing allergic disease risk profile [32, 33], these samples were excluded. This study received ethical approval from Health Canada, St. Justine’s Hospital (Montreal, QC), and the IWK Health Centre (Halifax, NS) and all participants provided consent.

### Environmental Contaminant Exposure

In the present investigation, four organochlorine pesticides, four PCB congeners, five organophosphate pesticide metabolites, and four metals were analyzed. These organochlorine pesticides and PCB congeners were measured in first trimester plasma (Table 1). The non-dioxin like PCB congeners 138, 153, and 180 were summed to create an index of PCB exposure. Plasma samples were analyzed by gas chromatography coupled to a mass spectrometer (Agilent 6890 N/5973 GC-MS). The organophosphate pesticide metabolites were measured in first trimester urine (Table 1). Index measures of the dimethyl alkylphosphate (DMAP) and diethyl alkylphosphate (DEAP) metabolites were calculated by summing the respective metabolites on a molar basis [34]. As only 2 % of samples had detectable concentrations of the organophosphate metabolite DEDTP, this metabolite was not analysed. Organophosphate metabolites in urine were measured with a GC-MS-MS instrument with a GC Agilent6890 N (Agilent Technologies; Mississauga, ON, Canada) coupled with a tandem mass spectrometer Quattro Micro GC (Waters; Milford, Massachusetts, USA).

**Table 1:** Descriptive statistics of environmental contaminants (MIREC study, 2008–2011)

**Chemical** ** (μg/L)**	**N**	**LOD**	**%** **>LOD**	**Min**	**Median**	**Max**	**IQR**
*Organochlorine Pesticides (plasma)*							
Trans-nonachlor	1232	0.01	84.58	LOD	0.02	0.23	0.01
p,p'-Dichlorodiphenyldichloroethylene (DDE)	1233	0.09	99.19	LOD	0.30	26.00	0.28
Hexachlorobenzene	1232	0.04	29.71	LOD	0.02	0.61	0.02
Oxychlordane	1231	0.005	92.04	LOD	0.01	0.13	0.01
*Organophosphate Pesticide Metabolites* *(urine*)^a^							
Dimethylphosphate (DMP)	1229	1.00	79.55	LOD	3.25	43.33	4.44
Dimethylthiphosphate (DMTP)	1227	0.60	80.49	LOD	3.44	141.82	7.22
Diethyldithiophosphate (DEDTP)	1230	0.30	2.27	LOD	0.15	4.02	0.18
Diethylthiophosphate (DETP)	1229	0.3,0.6	52.92	LOD	0.65	31.89	0.85
Diethylphosphate (DEP)	1230	1.00	76.64	LOD	2.38	2104.76	2.90
*Polychlorinated biphenyls (plasma)*							
PCB 118	1233	0.01	74.45	LOD	0.01	0.22	0.02
PCB 138	1233	0.01	93.19	LOD	0.03	0.28	0.02
PCB 153	1233	0.01	99.19	LOD	0.04	0.53	0.04
PCB 180	1233	0.01	92.70	LOD	0.03	0.77	0.03
*Metals (whole blood)*							
Lead (μg/dL)	1241	0.10	100.00	0.17	0.62	4.14	0.41
Cadmium	1241	0.04	97.42	LOD	0.20	5.06	0.17
Arsenic	1241	0.22	93.63	LOD	0.82	34.46	0.66
Mercury	1241	0.12,0.10	90.57	LOD	0.70	10.03	1.02

^a^adjusted for specific gravity [41]

First and third trimester whole blood metals (lead, mercury, arsenic, and cadmium) concentrations were averaged to create an index of exposure throughout pregnancy. Metal concentrations were analyzed using inductively coupled plasma mass spectrometry (PerkinElmer ELAN ICP-MS DRC II). All chemical analyses of blood and urine samples were carried out at the Toxicology Centre of the Quebec Institute of Public Health, accredited by the Standards Council of Canada.

### Fetal Immune System Markers

Immune system biomarkers were measured in the plasma of umbilical cord blood samples (details in [17]). TSLP concentrations were determined using a commercial antibody kit (Biolegend, San Diego, CA, USA). IL-33 concentrations were analyzed using antibodies from an R & D systems duoset (Minneapolis, MN, USA). ELISA kits (EBioscience, San Diego, CA, USA) were also used to assess both total IgE and IgA concentrations.

### Statistical Analysis

For the descriptive statistics, urinary concentrations of the organophosphate metabolites were adjusted for specific gravity to account for heterogeneity in urinary dilution according to the following formula P_c_ = P_i_ [(SG_m_- 1)/(SG_i_ - 1)], where: *P*_*c*_ = SG adjusted metabolite concentration (μg/ml), *P*_i_ = observed metabolite concentration,, *SG*_i_ = specific gravity of the urine sample, and SG_m_ = median SG for the cohort [35].

Due to the high percentage of cord blood samples below the limit of detection (LOD), each immune system biomarker was categorized into a binary variable. A composite variable was developed to identify samples with elevated concentrations of both TSLP and IL-33 (IL-33/TSLP) as these cytokines are highly correlated (Spearman correlation coefficient = 0.8) and known to interact in experimental models [26]. Elevated concentrations of TSLP and IL-33 were defined as samples that exceeded the 80^th^ percentile for both cytokines (TSLP = 554 pg/mL; IL-33 = 879 pg/mL) because there are no pre-existing thresholds. Elevated IgE samples were defined as those that exceeded 1.2 ng/mL (0.5 kU/L), a cut-off point previously used in studies of cord blood IgE [36, 37].

Logistic regression was employed to estimate the association between log-transformed continuous variables and elevated concentrations of IgE and IL-33/TSLP with the exception of hexachlorobenzene. Due to the high percentage of samples below the LOD for this chemical, a binary variable categorized at the LOD was created. For all other chemicals, maximum likelihood estimation was used, assuming a log-normal distribution for sample concentrations. This method of accounting for samples with values below the LOD is less prone to bias than substitution methods that use LOD/2 or LOD/√2 [38]. For environmental contaminants with 2 LODs (e.g. mercury, DETP), all values less than the higher LOD were treated as below the limit of detection. For the summary measures of PCBs congeners and organophosphate metabolites, a value was treated as less than the LOD if each of the individual metabolite values were undetectable.

Potential covariates were identified based on previous literature of predictors of the environmental contaminant exposures (organochlorine pesticides [39], organophosphorous pesticides [40], PCBs [41], metals [42–44]) and predictors of the immune system biomarkers [17, 45]. Maternal blood lipid concentrations were included in the organochlorine and PCB models to account for the lipophilic nature of these chemicals and intra-individual heterogeneity in lipids [46]. Maternal urinary specific gravity was included into the organophosphate pesticide metabolite models to account for heterogeneity in urinary dilution [47]. Maternal age was *a priori* entered into all models; no predictors of both exposure and outcome were identified. The linearity of the relationship between each environmental contaminant and immune system biomarker was assessed using restricted cubic splines [48]. To assess whether relationships differed by infant sex, the P-value of the sex*exposure interaction term was assessed and sex-stratified analyses were conducted. For statistically significant associations, two additional analyses were conducted using; i) tertiles of exposure and ii) a doubling of exposure concentrations. This was done for ease of interpretation and visualization of results. A linear test for trend was performed to evaluate dose–response relationships between tertiles of exposure and the immune system biomarker of interest; resulting P-values were reported.

## Results

Of the 2,001 women recruited, 18 withdrew and asked that all their data and biospecimens be destroyed. Of the remaining 1,983 subjects, 1,363 women had a cord blood sample. Of these 1,363 samples, 107 were excluded for missing chemical exposure data, a high IgA concentration, pre-term birth (<37 weeks), multiple birth, or samples with insufficient sample for analysis. Among the eligible subset of study participants (term, singleton births), there were no notable differences between women with and without a cord blood sample in regard to parental smoking, maternal age, or BMI. Seventy-three percent of the eligible participants in the cohort had a cord blood sample that was included in the analytical sample.

Table 1 shows descriptive statistics of the environmental contaminants. The percentage of samples above the limit of detection ranged from 29.7 % for hexachlorobenzene to 100 % for lead. Maternal demographic, reproductive, and infant characteristics are depicted in Table 2. The majority of study participants were 30 years or older, had a household income greater than $50,000, were non-smokers, and of normal BMI.

**Table 2:** Study participant characteristics, MIREC Study, Canada, 2008–2011 (n = 1256)^a^

Characteristic	N	%
*Maternal Demographic*		
Maternal Age (yrs.)		
≤24	60	4.8
25–29	270	21.5
30–34	450	35.8
≥35	476	37.9
Household Income ($CAD)		
≤30,000	90	7.5
30,001-50,000	116	9.6
50,001-100,000	514	42.6
>100,000	487	40.4
Parental Smoking ^b^		
No	1006	80.1
Yes	250	19.9
Pet Ownership		
No	559	44.5
Yes	697	55.5
*Maternal Reproductive & Medical History*		
Pre-Pregnancy BMI^c^		
Underweight (<18.5)	27	2.3
Normal (18.5 to 24.9)	717	60.5
Overweight (25 to 29.9)	272	22.9
Obese (≥30)	170	14.3
Maternal Allergy^d^		
No	1202	95.7
Yes	54	4.3
Parity		
Nulliparous	526	41.9
Primiparous	512	40.8
Multiparous	216	17.2
	2	
*Infant Characteristics*		
Infant Sex		
Male	671	53.5
Female	584	46.5
Birth Weight (g)		
<2500	11	0.9
2500- < 3500	606	48.2
3500- < 4000	448	35.7
≥4000	192	15.3

^a^Subtotals may not sum to total due to missing data, ^b^defined as either the mother or father smoking during pregnancy, ^c^World Health Organization Classification [66], ^d^Defined as maternal use of allergy medication

As no chemical met the criteria for non-linearity, all exposures were entered into the model as continuous variables without a quadratic term. Results of the IL-33/TSLP and IgE models are presented in Tables 3 and 4 respectively. All models were adjusted for age and, depending on the specific chemical, specific gravity or lipids. Significant inverse associations were observed between maternal lead concentrations, DDE concentrations, PCB 118 concentrations, ∑DEP and DETP concentrations and elevated concentrations of IL-33/TSLP (lead aOR = 0.70 95 % CI: 0.48-0.94; DDE aOR = 0.81 95 % CI: 0.63-0.98; PCB 118 aOR = 0.69 95 % CI: 0.40-0.98; ∑DEP and DETP aOR = 0.85 95 % CI: 0.72-0.97). No significant associations were observed between any of the environmental contaminants and elevated cord blood concentrations of IgE. There was no observed effect modification by sex or material differences in the sex-stratified analysis (data not shown).

**Table 3:** Odds ratio of log_10_ maternal contaminant concentrations and elevated (≥80 %) cord blood concentrations of IL-33/TSLP

**Chemical ** **(μg/L)**	**N**	**Unadjusted ** **OR** ** (95 % CI)**	**Adjusted ** **OR** ** (95 % CI)**
*Organochlorine Pesticides* ^**a**^			
Trans-nonachlor	1232	0.83 (0.61-1.07)	0.77 (0.46-1.08)
DDE	1233	0.80 (0.64-0.97)	0.81 (0.63-0.98)
Hexachlorobenzene (binary)	1232	0.79 (0.56-1.10)	0.79 (0.56-1.14)
Oxychlordane	1231	0.84 (0.61-1.07)	0.80 (0.49-1.10)
*Organophosphate Pesticide Metabolites* ^*b*^			
Dimethylphosphate (DMP)	1229	0.86 (0.75-0.97)	0.85 (0.68-1.02)
Dimethylthiphosphate (DMTP)	1227	0.99 (0.89-1.08)	0.98 (0.84-1.12)
Diethylthiophosphate (DETP)	1229	0.95 (0.81-1.09)	0.96 (0.72-1.22)
Diethylphosphate (DEP)	1230	0.85 (0.73-0.97)	0.87 (0.67-1.06)
ƩDMP&DMTP	1226	0.90 (0.79-1.02)	0.91 (0.80-1.03)
ƩDEP&DETP	1229	0.85 (0.72-0.98)	0.85 (0.72-0.97)
*Polychlorinated biphenyls* ^*a*^			
PCB 118	1233	0.83 (0.62-1.05)	0.69 (0.40-0.98)
PCB 138	1233	0.85 (0.67-1.05)	0.86 (0.62-1.10)
PCB 153	1233	0.87 (0.68-1.06)	0.86 (0.65-1.08)
PCB 180	1233	0.87 (0.70-1.04)	0.95 (0.72-1.19)
ƩPCB (non-dioxin – 138,153,180)	1233	0.87 (0.68-1.07)	0.89 (0.67-1.11)
*Metals* ^**c**^			
Lead	1241	0.70 (0.48-0.94)	0.72 (0.48-0.95)
Cadmium	1241	0.91 (0.73-1.10)	0.92 (0.73-1.11)
Arsenic	1241	0.94(0.73-1.14)	0.95 (0.73-1.17)
Mercury	1241	0.94 (0.80-1.07)	0.96 (0.82-1.10)

^a^adjusted for age and lipids, ^b^adjusted for age and specific gravity, ^c^adjusted for age

Table 5 depicts the results of the tertile analysis for chemicals with significant associations in the analyses using continuous exposures. Results were consistent with the analysis of continuous exposures though none of the effect measures achieved statistical significance. A suggestion of an inverse finding was observed in the relationship between both DDE and PCB 118 and IL-33/TSLP (P-value test for trend = 0.06 for both contaminants). A two fold increase in log maternal lead, PCB 118, DDE and ∑DEP and DETP concentrations was associated with odds ratios comparable to the analysis using continuous exposures and tertiles (lead aOR = 0.80 95 % CI:0.63-1.00; DDE aOR =0.86 95 % CI:0.74-1.00; PCB 118 aOR = 0.78 95 % CI:0.58-1.03; ∑DEP and DETP aOR = 0.91 95 % CI: 0.80-1.04).

**Table 4:** Odds ratio of log_10_ maternal contaminant concentrations and elevated (≥0.5 ku/L) cord blood concentrations of IgE

**Chemical ** **(μg/L)**	**N**	**Unadjusted ** **OR** ** (95 % CI)**	**Adjusted ** **OR** ** (95 % CI)**
*Pesticides*			
*Organochlorine Pesticides* ^**a**^			
Trans-nonachlor	1232	0.81 (0.59-1.03)	0.86 (0.51-1.10)
DDE	1233	0.93 (0.75-1.12)	1.00 (0.79-1.20)
Hexachlorobenzene (binary)	1232	0.87 (0.62-1.20)	0.89 (0.62-1.27)
Oxychlordane	1231	0.84 (0.61-1.07)	1.01 (0.62-1.40)
*Organophosphate Pesticide Metabolites* ^*b*^			
Dimethylphosphate (DMP)	1229	0.97 (0.85-1.10)	1.01 (0.81-1.21)
Diethylphosphate (DEP)	1230	0.91 (0.78-1.04)	0.88 (0.69-1.08)
Dimethylthiphosphate (DMTP)	1227	0.98 (0.88-1.07)	0.92 (0.79-1.06)
Diethylthiophosphate (DETP)	1229	0.99 (0.84-1.13)	0.95 (0.70-1.19)
ƩDMP&DMTP	1226	0.96 (0.84-1.08)	1.00 (0.81-1.11)
ƩDEP&DETP	1229	0.95 (0.81-1.09)	0.86 (0.70-1.01)
*Polychlorinated biphenyls* ^*a*^			
PCB 118	1233	0.98 (0.74-1.23)	1.08 (0.68-1.49)
PCB 138	1233	0.96 (0.75-1.17)	1.11 (0.81-1.40)
PCB 153	1233	0.96 (0.76-1.17)	1.08 (0.82-1.33)
PCB 180	1233	0.93 (0.75-1.11)	1.07 (0.82-1.33)
ƩPCB (non-dioxin – 138,153,180)	1233	0.95 (0.74-1.16)	1.07 (0.81-1.33)
*Metals* ^**c**^			
Lead	1241	0.92 (0.63-1.23)	0.98 (0.66-1.30)
Cadmium	1241	0.90 (0.72-1.09)	0.91 (0.73-1.10)
Arsenic	1241	1.16 (0.90-1.41)	1.20 (0.93-1.47)
Mercury	1241	0.95 (0.81-1.09)	1.00 (0.84-1.15)

^a^adjusted for age and lipids, ^b^adjusted for age and specific gravity, ^c^adjusted for age

**Table 5:** Odds ratios of tertiles of exposure and elevated (≥80 %) cord blood concentrations of IL-33/TSLP for contaminants shown to have significant associations from Table 2

Contaminant	Tertiles	Adjusted OR (95 % CI)
DDE^a^ (ug/L)	≤0.23	1.0
(n = 1233)	0.24-0.39	0.75 (0.51-1.09)
	>0.39	0.69 (0.47-1.02)
P-value test for trend		0.06
PCB 118^a^ (ug/L)	≤0.011	1.0
(n = 1233)	0.011-0.018	0.85 (0.58-1.23)
	>0.018	0.67 (0.45-1.02)
P-value test for trend		0.06
ƩDEP&DETPb^b^(ug/L)	≤12.60	1.0
(n = 1229)	12.61-26.90	0.84 (0.57-1.24)
	>26.90	0.89 (0.56-1.40)
P-value test for trend		0.08
Lead^c^ (ug/dL)	≤0.68	1.0
(n = 1241)	0.69-1.08	0.83 (0.57-1.20)
	>1.08	0.77 (0.53-1.11)
P-value test for trend		0.16

^a^adjusted for age and lipids, ^b^adjusted for age and specific gravity, ^c^adjusted for age

## Discussion

In this study, we sought to characterize the association between prenatal exposure to PCBs, pesticides and metals and elevated concentrations of IgE, IL-33 and TSLP. We observed inverse associations between maternal lead, PCB 118, DDE, ∑DEP and DETP and IL-33/TSLP. No effect modification by sex was observed in these relationships. Our findings suggest that contemporary maternal concentrations of these environmental contaminants are not associated with developmental immunotoxicity as measured by the immune system biomarkers of interest.

Experimental evidence has demonstrated that gestational lead exposure may elevate IgE concentrations [3, 49] and enhance Th2 cell responses [50]. A significantly elevated risk of childhood atopy was observed among children with elevated maternal and cord blood lead concentrations in a Polish birth cohort [51]. Significant correlations between childhood lead concentrations and both elevated IgE [52–54] and atopic dermatitis [55] have been identified in cross-sectional analyses. In light of these previous findings, the observed lack of association between lead and IgE an inverse association between lead and IL-33/TSLP in the present study was unexpected.

There is experimental evidence that PCBs may enhance production of Th2 cell cytokines involved in allergic responses [8]. Similar associations have not, however, been consistently observed in epidemiologic studies. For example, a meta-analysis of ten European cohort studies concluded that prenatal PCB-153 exposure was not associated with infant wheeze or bronchitis [56]. Conversely, authors of a recent Dutch cohort study reported a positive association between prenatal PCB-153 exposure and self-reported asthma medication use between the ages of 6 and 20 [57].

Experimental evidence has also demonstrated that organochlorine pesticide exposure, specifically DDE, may also induce allergic type responses via enhanced production of Th2 cytokines [6]. Though positive associations between prenatal DDE exposure and measures of childhood allergic disease have been reported in two birth cohort studies [58, 59], a meta-analysis of 10 cohort studies reported that this association was limited to children less than 18 months old [56]. In addition, authors of the Dutch cohort study reported no significant association between prenatal DDE exposure (median = 2.47 ng/mL) and asthma medication use [57]. While organophosphorous pesticides may alter immune system function [6], we did not identify any birth cohort studies of maternal organophosphorous pesticides exposures and childhood allergic outcomes.

In light of this literature, two primary factors may explain the present findings. First, compared to historical cohorts [51, 57], maternal environmental contaminant concentrations in the MIREC study are relatively low. Average lead concentrations in the MIREC study (geometric mean = 0.88 μg/dL) were lower than observed in the Polish birth cohort (geometric mean = 1.60 μg/dL) [51] and notably lower than 1 to 2 ug/dL, values which have been reported to be associated with adverse health effects [60]. Maternal concentrations of PCB-153 and DDE were also lower among MIREC participants than in the Dutch cohort study (PCB-153 median = 1.37 μg/L, DDE median = 0.3 μg/L) [57]. Second, it possible that the observed inverse associations may be a product of live birth bias [61]. Fetuses that were most susceptible to the toxic effects of contaminant exposure may not have survived until birth. The term fetuses in the present analysis may, therefore, represent a ‘healthy’ subset of the total number of pregnancies among MIREC study participants.

Our study was also subject to the following four limitations. First, although the persistent organic pollutants and metals have long half-lives and, therefore, little expected variation throughout pregnancy, the organophosphorous metabolites have relatively short half-lives [62] and may vary during pregnancy [63]. Furthermore, first trimester urinary measures may not be representative of exposure during critical windows of immune system development. Considering that 1) that exposure misclassification is non-differential and 2) this study employed continuous rather than polytomous exposure variables, any potential misclassification bias likely attenuated effect estimates towards the null [64]. Second, due to the limited literature regarding IL-33 and TSLP at birth, our choice of cut-off points was not based on clinical guidelines. Rather, we based this cut-off on our previous analysis of predictors of elevated concentrations of IL-33 and TSLP at birth [17]. In the present analysis, we conducted a sensitivity analysis for chemicals with statistically significant associations by dichotomizing IL-33 and TSLP at the limit of detection. As we observed similar odds ratios when using the limit of detection cut-off, we have only reported results using the 80^th^ percentile cut-off. Third, as we did not have clinical data on asthma development in the children, it is not possible to determine the clinical implications of our findings. Fourth, the present results may be subject to negative confounding by an unmeasured protective factor for allergy, such as maternal vitamin D [65].

Despite these limitations, this study benefited from the relatively large sample size and the comparatively rich covariate data. Considering the relatively long-half life for the persistent organic pollutants, there is unlikely to be considerable within-person variability in exposure throughout pregnancy. The value of the present research will become most evident when combined with follow-up studies of MIREC participants into childhood. In conjunction with such follow-up, this research will help determine the timing of any potential immunotoxic effect of in utero exposures and may, ultimately, strengthen understanding of the developmental origins of childhood allergy. This information is relevant to identifying critical windows of exposure and ultimately, to developing public health messages regarding minimizing environmental contaminant exposure.

## Conclusions

In this primarily urban Canadian population of pregnant women and their newborns, maternal concentrations of persistent organic pollutants, pesticides, and metals were not associated with elevated IgE, TSLP or IL-33. Generalization of these findings beyond the MIREC study population would require examination in a population of differing socioeconomic status, ethnicity, or health profiles (e.g. smoking and obesity rates). Future studies that examine the trajectory of these biomarkers from birth to childhood will complement the present findings.

## Abbreviations

CV: coefficient of variation
DDE: p,p'-Dichlorodiphenyldichloroethylene
DEAP: diethyl alkylphosphate
DEDTP: diethyldithiophophate
DEP: diethylphosphate
DMAP: dimethyl alkylphosphate
DETP: diethylthiophosphate
DETP: diethylthiophosphate
DMP: Dimethylphosphate
DMTP: dimethylthiphosphate
ELISA: enzyme linked immunoassay
IgA: immunoglobulin A
IgE: immunoglobulin E
IL-33: interleukin-33
LOD: limit of detection
MIREC: Maternal-Infant Research on Environmental Chemicals
PCB: polychlorinated biphenyl
POP: persistent organic pollutant
SG: specific gravity
Th2: T helper 2
TSLP: thymic stromal lymphopoietin
